# First person – Kaitly Woodard

**DOI:** 10.1242/dmm.049644

**Published:** 2022-07-07

## Abstract

First Person is a series of interviews with the first authors of a selection of papers published in Disease Models & Mechanisms, helping early-career researchers promote themselves alongside their papers. Kaitly Woodard is first author on ‘ Limitations of mouse models for sickle cell disease conferred by their human globin transgene configurations’, published in DMM. Kaitly conducted the research described in this article while a graduate student in Mitchell J. Weiss's lab at St. Jude Children's Research Hospital, Memphis, TN, USA. She is now a Senior Scientist II in the lab of Mir Hossain at Novartis Institutes for BioMedical Research, Cambridge, MA, USA, investigating gene therapy for haemoglobinopathies.


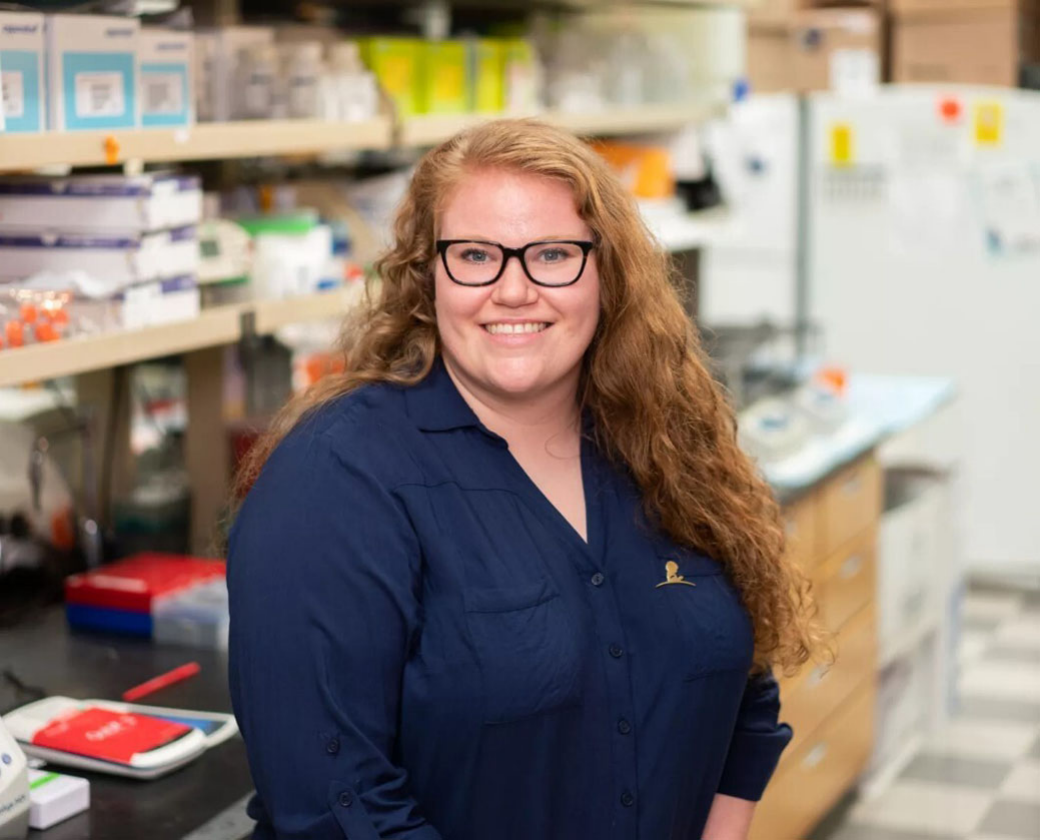


**Kaitly Woodard**

**How would you explain the main findings of your paper to non-scientific family and friends?**

The two most commonly used mouse models for sickle cell disease (SCD) were created in the late 1990s (Berkeley) and early 2000s (Townes) to recreate and study the disease. These mice are outdated; their design was based on what was known at the time and created using imprecise methods. Now, when we try to use the most cutting-edge technology like gene editing, we find challenges with using these older models. Our paper looks closely at the genetics of the two models available and identifies some of the limitations they pose. The Berkeley model was made by random insertion of a human sequence that we found inserted in the genome many times and being incompatible with gene editing. While the Townes mouse performs better, we sequenced the SCD locus insertion and found that some important sequences were left out – likely because they were not known to be important at the time. Ultimately, we provide a resource to help other labs identify which model may be useful for their research question, and to challenge investigators to reassess the limitations of older disease models.

“[…] we provide a resource to […] challenge investigators to reassess the limitations of older disease models.”

**What are the potential implications of these results for your field of research?**

Our paper will help investigators decide whether or not the current mouse models of SCD are fitting for the study they want to do, saving time and valuable resources. I hope this work spreads further than the field of haematology, to encourage researchers to look more critically at the design and development of older mouse models when trying to apply the newest technology.

**What are the main advantages and drawbacks of the model system you have used as it relates to the disease you are investigating?**

The multiple, identical genetic sequences present in the Berkeley model are incompatible with DNA double-strand break-induced gene-editing technology. Our CRISPR/Cas9 strategy to target a repressor-binding site at the globin locus has been shown to be therapeutic in human cells. However, the Berkeley SCD model has multiple copies of the target site and our attempts to reproduce the strategy result in cell toxicity and death because of the large number of DNA breaks.

The Townes model does not have the multiple copy number complication; however, its knock-in design left out many of the (recently identified) crucial regulatory elements responsible for regulating gene expression. When we applied our CRISPR/Cas9 strategy to cells from these mice, we saw less toxicity but no therapeutic effect like we have seen in human cells. We believe it is the missing regulatory elements that prevent sufficient and therapeutic gene expression.

**Figure:**
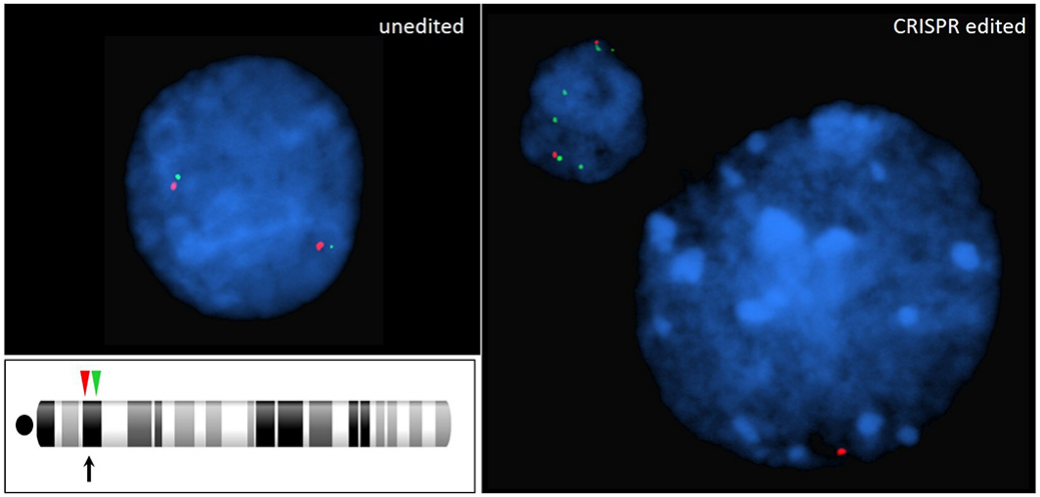
**Fluorescence *in situ* hybridization of interphase Berkeley mouse cells showing chromosomal abnormalities after CRISPR/Cas9 editing within the multi-copy human transgene.** Here we see an unedited cell (left) compared to a cell 24 h after CRISPR/Cas9 editing (right). The red (centromeric) and green (telomeric) probes flank the transgene insertion site (black arrow), and are normally clustered together as seen in the unedited cell. After editing, we see many signs of chromosomal abnormalities in the segregation of a chromosome arm at the cut site (between red and green probes) and the formation of a micronucleus (small nuclear structure formed around missegregated chromosomes). Overall, this is a dramatic example of the consequences of too many DNA double-strand breaks due to Cas9 editing within a repetitive region.

**What has surprised you the most while conducting your research?**

In a disease area like SCD, many research labs have used the same disease models for 20+ years. Despite their usefulness, I was surprised to find how little information on the human sequence within these models is published. In trying to clarify the genetic profile of the Berkeley and the Townes mice, I found limited publications, which spurred my desire to perform additional genetic characterization.

**Describe what you think is the most significant challenge impacting your research at this time and how will this be addressed over the next 10 years?**

I don't think either of the two commonly used SCD mouse models are ideal for studies that cover a range of research questions. As it stands right now, jumping between models depending on your research question may be the best experimentally, but can provide a strain on lab resources and finances. I hope that a new (or updated) model of SCD with a single copy of the human globin sequences involved in globin regulation will be developed using the most up-to-date technology. If done optimally, haemoglobin regulation and SCD could be studied within the same model with fewer of the limitations mentioned in the paper.

**What changes do you think could improve the professional lives of early-career scientists?**

I think that researchers should have more of a platform for sharing ‘negative’ results. For example, this paper represents many years of attempting CRISPR/Cas9 editing in the Berkeley transgene – a technique which we now know is incompatible within that mouse model. I am confident that previous investigators in the field either tried similar experiments or had ‘suspicions’ about the model's limitations that were never made public. This could have been alleviated by encouraging and giving researchers a better way to share such findings.

“I think that researchers should have more of a platform for sharing ‘negative’ results.”

**What's next for you?**

After defending my PhD dissertation in 2021, I accepted a Senior Scientist II position at Novartis Institutes for BioMedical Research (NIBR) in Cambridge, MA, USA, to pursue SCD gene-therapy projects and advance them towards the clinic. I plan to use my expertise in SCD and disease models to ensure that we're asking the right questions and using the best model to answer them.
